# Interaction of Orexin and Bone Morphogenetic Proteins in Steroidogenesis by Human Adrenocortical Cells

**DOI:** 10.3390/ijms241612559

**Published:** 2023-08-08

**Authors:** Yoshiaki Soejima, Nahoko Iwata, Ran Nishioka, Mako Honda, Yasuhiro Nakano, Koichiro Yamamoto, Atsuhito Suyama, Fumio Otsuka

**Affiliations:** Department of General Medicine, Okayama University Graduate School of Medicine, Dentistry and Pharmaceutical Sciences, 2-5-1 Shikata-cho, Kitaku, Okayama 700-8558, Japanasuyama@s.okayama-u.ac.jp (A.S.)

**Keywords:** bone morphogenetic protein (BMP), orexin, steroidogenesis and adrenal

## Abstract

Orexins are neuropeptides that play important roles in sleep-wake regulation and food intake in the central nervous system, but their receptors are also expressed in peripheral tissues, including the endocrine system. In the present study, we investigated the functions of orexin in adrenal steroidogenesis using human adrenocortical H295R cells by focusing on its interaction with adrenocortical bone morphogenetic proteins (BMPs) that induce adrenocortical steroidogenesis. Treatment with orexin A increased the mRNA levels of steroidogenic enzymes including StAR, CYP11B2, CYP17, and HSD3B1, and these effects of orexin A were further enhanced in the presence of forskolin. Interestingly, orexin A treatment suppressed the BMP-receptor signaling detected by Smad1/5/9 phosphorylation and Id-1 expression through upregulation of inhibitory Smad7. Orexin A also suppressed endogenous BMP-6 expression but increased the expression of the type-II receptor of ActRII in H295R cells. Moreover, treatment with BMP-6 downregulated the mRNA level of OX1R, but not that of OX2R, expressed in H295R cells. In conclusion, the results indicate that both orexin and BMP-6 accelerate adrenocortical steroidogenesis in human adrenocortical cells; both pathways mutually inhibit each other, thereby leading to a fine-tuning of adrenocortical steroidogenesis.

## 1. Introduction

Orexins are neuropeptides that have various effects on sleep and wakefulness regulation, food intake, emotions, and stress responses [1,2]. Orexins have two isoforms, orexin A and orexin B, which are derived from a precursor peptide [1]. Orexins act through two types of G protein-coupled receptors: orexin receptor type 1 (OX1R) and orexin receptor type 2 (OX2R). Orexin A binds to both OX1R and OX2R, whereas orexin B binds only to OX2R [1,2,3]. Orexins and their receptors are not restricted to the hypothalamus but are also expressed in peripheral tissues such as the pancreas, gonads, kidney, intestine, adipose tissue, and adrenal gland [4]. It has been confirmed that orexins affect pancreatic insulin and glucagon secretion, testicular androgen production, ovulation, intestinal motility and secretion, lipid metabolism, and adrenomedullary catecholamine release [4]. Furthermore, there is growing evidence that orexins play functional roles in the regulation of the endocrine system, including the hypothalamic-pituitary-adrenal (HPA) axis [1,5,6].

It has been reported that OX1R and OX2R are expressed in the rat, porcine, and human adrenal cortex [7,8] and that orexins stimulate glucocorticoid and mineralocorticoid secretion from adrenocortical cells of various species [9,10,11,12,13,14]. Importantly, there is accumulating evidence that orexins are involved in the control of blood pressure and hypertension [15,16]. Pharmacological blockade of orexin receptors reduced blood pressure in spontaneously hypertensive rats, an animal model of human essential hypertension [17], and in BPH/2J mice, a genetic model of hypertension associated with an overactive sympathetic nervous system [18]. The upregulation of OX1R contributed to hypertension in obese Zucker rats, an animal model of obesity-related hypertension [19]. In addition, studies on orexin-deficient narcoleptic patients and animal models showed a variable decrease in arterial blood pressure (ABP) during wakefulness and a blunted decrease in ABP from wakefulness to non-rapid-eye-movement sleep (NREM) and rapid-eye-movement sleep (REM) [20,21]. Of note, there have been a few studies in which the HPA system in individuals with narcolepsy was investigated. It was found that the basal secretion of adrenocorticotropin (ACTH) was dramatically reduced [22] and that cortisol levels after dexamethasone suppression were significantly lowered in narcolepsy patients [23]. These results suggested that orexin is physiologically involved in the control of blood pressure via various mechanisms, which potentially include the regulation of the HPA axis and adrenal steroids.

Adrenocortical steroidogenesis is directly stimulated by angiotensin II (Ang II), ACTH, and potassium. In addition to the major stimulants, several factors have been reported to play roles in the regulation of adrenocortical steroidogenesis [24,25]. Among these, we have focused on the roles of bone morphogenetic proteins (BMPs), which are members of the transforming growth factor (TGF)-β superfamily, in the adrenal gland [26]. The BMP receptors consist of type-I receptors, including activin receptor-like kinase (ALK)-2, -3, and -4, and type-II receptors, including activin type-II receptor (ActRII) and BMP type-II receptor (BMPRII) in human adrenocortical cells [27]. Based on the results of in vitro studies, BMP-6 enhances Ang II-induced aldosterone production via activation of mitogen-activated protein kinase (MAPK) signaling by interacting with ALK-2 and -3, and ActRII among the BMP receptors [27,28,29,30]. In addition, activin enhances ACTH-induced aldosterone production by activating cyclic adenosine monophosphate (cAMP)/protein kinase A (PKA) signaling [27,31]. Interestingly, it has recently been found that melatonin, a pineal gland hormone that is involved in sleep regulation and circadian functions [32], enhanced aldosterone production induced by ACTH, and activin [31] and that Clock mRNA expression was suppressed by both BMP-6 and activin and was linked to the expression of steroidogenic enzymes [33]. However, the functional link between the signaling of BMPs and orexin in adrenocortical steroidogenesis has not been elucidated.

In the present study, we aimed to investigate the functional roles of orexin in the regulation of adrenocortical steroidogenesis by focusing on its interaction with the BMP system in human adrenocortical cells. 

## 2. Results

First, the effects of orexin on adrenocortical steroidogenesis were examined. Since there have been some studies showing that changes in the expression of steroidogenic enzymes and the concentration of secreted cortisol after orexin treatment were time-dependent, with 24 h treatment exhibiting one of the peaks [34,35], the treatment time was fixed at 24 h. Forskolin (FSK) is a stimulator of adenylyl cyclase and increases intracellular cAMP, which leads to the induction of steroidogenesis in the adrenal cortex [36]. Given the findings that an adenylyl cyclase inhibitor and a PKA inhibitor abolished the enhanced cortisol secretion by orexin A [37], the cAMP/PKA pathway is possibly linked to the roles of orexin A in adrenocortical steroidogenesis. Moreover, it has been recently demonstrated that HSD3B1 is expressed in the zone glomerulosa and is stimulated by Ang II rather than potassium [38,39]. 

**Figure 1:**
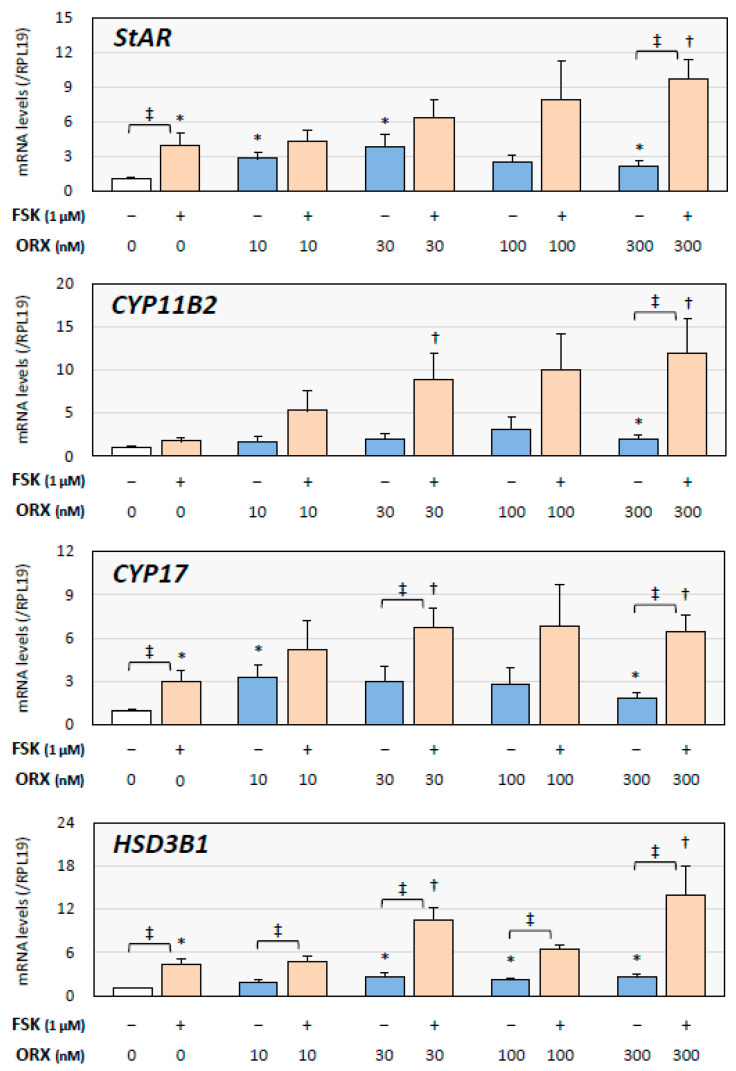
Effects of orexin A and forskolin on the expression of steroidogenic enzymes by human adrenocortical cells. H295R cells (3 × 10^5^ cells/mL) were treated with orexin A (ORX; 10–300 nM) in the presence or absence of forskolin (FSK; 1 μM) in serum-free DMEM/F12 for 24 h. Total cellular RNAs were extracted and the mRNA levels of steroidogenic enzymes, StAR, CYP11B2, CYP17, and HSD3B1, were standardized by RPL19 mRNA levels and expressed as fold changes. Results are shown as means ± SEM and were analyzed by the unpaired *t*-test; *, *p* < 0.05 vs. basal groups; †, *p* < 0.05 vs. FSK alone groups; ‡, *p* < 0.05 between the indicated groups.

As shown in Figure 1, orexin A (10 to 300 nM) treatment significantly enhanced the expression of steroidogenic enzymes, including StAR, CYP11B2, CYP17, and HSD3B1, in the absence of FSK. However, the orexin A concentration showing the maximal effect was different among the steroidogenic enzymes: 30 nM of orexin A for the expression of StAR, 100 nM for that of CYP11B2, 10 nM for that of CYP17, and 300 nM for that of HSD3B1. FSK (1 μM) stimulation increased the expression of steroidogenic enzymes, and co-treatment with orexin A further upregulated the expression of the enzymes in a dose-dependent manner. Co-treatment with 300 nM of orexin A resulted in a significant enhancement of the expression of all of the enzymes. Thus, orexin treatment enhanced the expression of steroidogenic enzymes and enhanced the FSK-induced expression of steroidogenic enzymes in H295R cells. 

**Figure 2:**
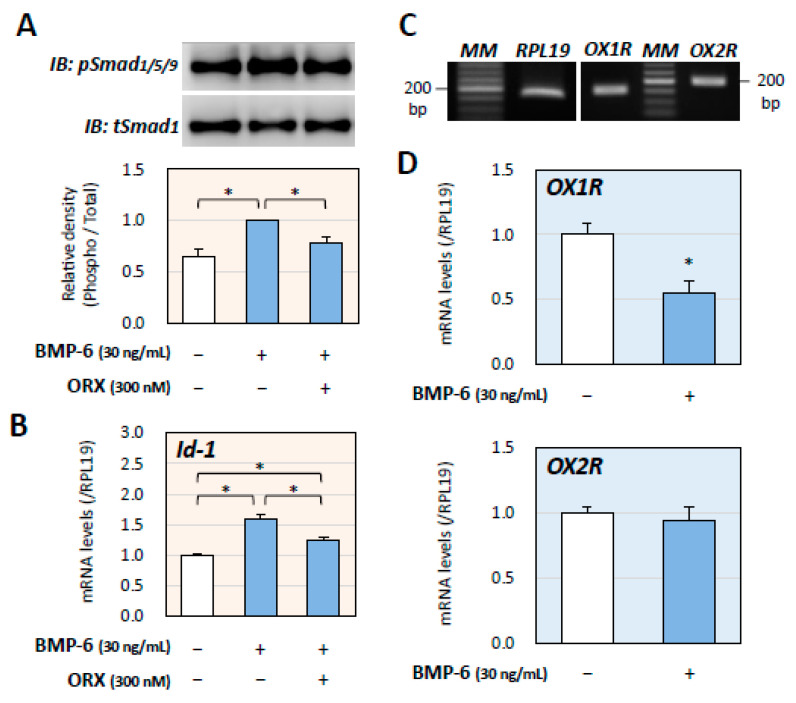
Mutual interaction of the signaling of orexin A and BMP in adrenocortical cells. (**A**) H295R cells (3 × 10^5^ cells/mL) were pretreated with orexin A (ORX; 300 nM) in serum-free DMEM/F12 for 24 h. After 1 h stimulation with BMP-6 (30 ng/mL), the cells were lysed and then subjected to immunoblot (IB) analysis by using antibodies that detect pSmad1/5/9 and tSmad1. The integrated signal density of each protein band was digitally analyzed and the ratios of signal intensities of pSmad1/5/9/tSmad1 were calculated. The results are representative of those obtained from at least three independent experiments and are expressed as fold changes. Results are shown as means ± SEM and were analyzed by the unpaired *t*-test. (**B**) H295R cells (3 × 10^5^ cells/mL) were treated with BMP-6 (30 ng/mL) with or without ORX (300 nM) in serum-free DMEM/F12 for 24 h. Total RNAs were extracted and the mRNA levels of Id-1 were standardized by RPL19 levels and expressed as fold changes. Results are shown as means ± SEM and were analyzed by the unpaired *t*-test. (**C**) The expression of mRNAs encoding OX1R, OX2R, and PRL19 was examined by RT-PCR in H295R cells. (**D**) H295R cells (3 × 10^5^ cells/mL) were treated with BMP-6 (30 ng/mL) in serum-free DMEM/F12 for 24 h. Total RNAs were extracted and the mRNA levels of OX1R and OX2R were standardized by RPL19 levels and expressed as fold changes. Results are shown as means ± SEM and were analyzed by the unpaired *t*-test; * *p* < 0.05 vs. basal groups and between the indicated groups.

Next, the effects of orexin on BMP signaling, an inducer of adrenocortical steroidogenesis, were evaluated using H295R cells. Stimulation with BMP-6 (30 ng/mL) for 1 h readily activated the phosphorylation of Smad1/5/9 in H295R cells, and pretreatment with orexin A (300 nM) for 24 h suppressed the Smad1/5/9 phosphorylation induced by BMP-6 (Figure 2A). Then, the effect of orexin A on the transcription of Id-1, a target gene of BMP signaling, was examined. As shown in Figure 2B, BMP-6 (30 ng/mL) treatment for 24 h enhanced Id-1 mRNA expression, whereas co-treatment with orexin A (300 nM) suppressed the expression of Id-1 induced by BMP-6. These results suggested that orexin A suppressed BMP signaling in H295R cells. 

We then confirmed the mRNA expression of OX1R (158 bp) and OX2R (204 bp) in H295R cells (Figure 2C). Thus, the effects of BMP-6 on orexin receptors were examined. It was found that treatment with BMP-6 (30 ng/mL) for 24 h significantly downregulated the expression of OX1R but not that of OX2R (Figure 2D). These results suggested that BMP-6 suppressed the effects of orexin A on H295R cells by downregulating OX1R expression.

**Figure 3:**
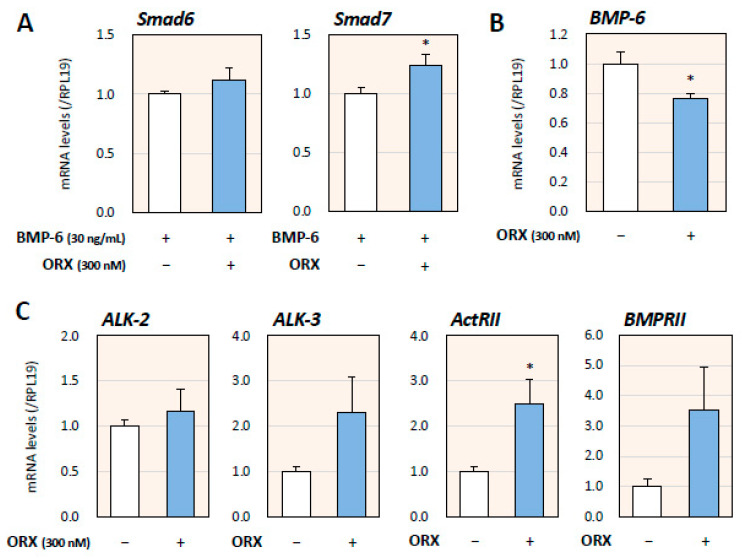
Effects of orexin A on expression of BMP signaling and endogenous BMP-6 in adrenocortical cells. (**A**) H295R cells (3 × 10^5^ cells/mL) were treated with BMP-6 (30 ng/mL) with or without orexin A (ORX; 300 nM) in serum-free DMEM/F12 for 24 h. Total cellular RNAs were extracted and the mRNA levels of Smad6 and Smad7 were standardized by RPL19 levels and expressed as fold changes. Results are shown as means ± SEM and were analyzed by the unpaired *t*-test; * *p* < 0.05 vs. basal groups. (**B**,**C**) H295R cells (3 × 10^5^ cells/mL) were treated with orexin A (ORX; 300 nM) in serum-free DMEM/F12 for 24 h. Total RNAs were extracted and the mRNA levels of BMP-6 (**B**) and BMP receptors (ALK-2, -3, ActRII, and BMPRII; (**C**)) were standardized by RPL19 levels and expressed as fold changes. Results are shown as means ± SEM and were analyzed by the unpaired *t*-test; * *p* < 0.05 vs. basal groups.

To investigate the mechanism by which orexin A suppresses BMP signaling in H295R cells, we further examined the effects of orexin A on BMP signaling, endogenous BMP-6, and BMP receptors. It was found that orexin A (300 nM) treatment for 24 h upregulated the expression of Smad7 (inhibitory Smad; I-Smad) in the presence of BMP-6 (30 ng/mL), suggesting that orexin suppresses BMP signaling by activating I-Smad (Figure 3A). Orexin A treatment (300 nM) for 24 h suppressed the expression of endogenous BMP-6 mRNA in H295R cells (Figure 3B). On the other hand, orexin A (300 nM) tended to upregulate the expression of ALK-3, ActRII, and BMPRII, in which the expression of ActRII was significantly upregulated among the BMP receptor subtypes, suggesting that downregulation of BMP signaling by orexin A enhances the expression of BMP receptors as a feedback loop (Figure 3C).

## 3. Discussion

**Figure 4:**
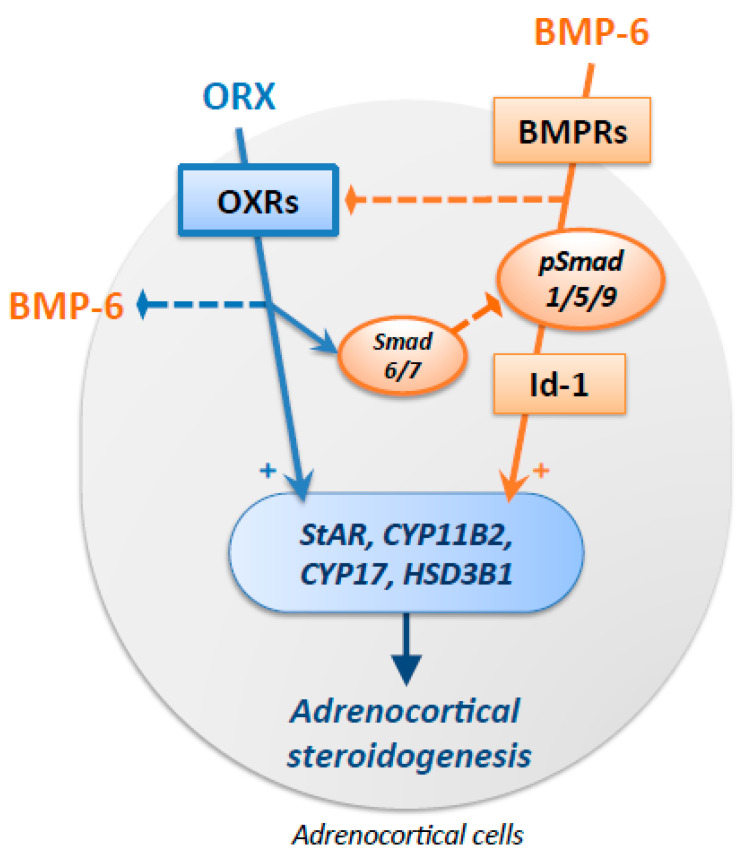
Functional interaction of the signaling of orexin A and BMP in adrenocortical cells. Orexin A (ORX) stimulated the expression of steroidogenic enzymes, including StAR, CYP11B2, CYP17, and HSD3B1. ORX further upregulated the expression of these steroidogenic enzymes stimulated by forskolin-induced activation of adenylyl cyclase. ORX suppressed BMP-6-induced Smad1/5/9 phosphorylation and Id-1 mRNA expression via the upregulation of Smad6/7. ORX suppressed the expression of endogenous BMP-6. On the other hand, BMP-6 downregulated the expression of orexin receptors (OXRs). Thus, functional interactions between the signaling of orexin A and BMP for the regulation of adrenocortical steroidogenesis were shown in adrenocortical cells. Dotted lines indicate inhibitory actions.

In the present study, the roles of orexin A and BMP signaling in adrenocortical steroidogenesis were uncovered in human adrenocortical H295R cells (Figure 4). Orexin A enhanced the expression of steroidogenic enzymes and further upregulated the expression of these enzymes induced by FSK. Orexin A suppressed both Smad1/5/9 phosphorylation and subsequent Id-1 mRNA expression via upregulation of I-Smad. Orexin A suppressed the expression of endogenous BMP-6 and enhanced the expression of ActRII among the BMP receptor subtypes, indicating that orexin A suppresses BMP-Smad signaling and induces BMP receptor expression, possibly as a feedback system. On the other hand, BMP-6 treatment downregulated OX1R expression. These results suggest that orexin A and BMP, both of which stimulate adrenocortical steroidogenesis, mutually suppress the downstream signaling, leading to the fine-tuning of adrenocortical steroidogenesis in adrenocortical cells. 

In this present study, orexin upregulated the expression of StAR, CYP11B2, CYP17, and HSD3B1, indicating an increased production of both mineralocorticoids and glucocorticoids. Consistent with these results, it was reported that orexins increased the mRNA levels and protein levels of StAR in H295R cells in a dose-dependent manner and that the effects were blocked by treatment with an OX1R antagonist [34]. It was also shown that orexins increased the promoter activity of CYP11B2, 3β-hydroxysteroid dehydrogenase type 2 (HSD3B2), and to a lesser extent, CYP11B1 and human steroid 21-hydroxylase (CYP21) [40]. Moreover, it was reported that orexin A increased the mRNA and protein expression levels of 3β-HSD and cortisol secretion by H295R cells in a dose-dependent manner, with 1 μM of orexin A showing the maximal effect. Of note, the effects were partly blocked by an OX1R antagonist [41,42]. These results suggest that orexin enhances a wide range of steroidogenic enzymes that are involved in the synthesis of mineralocorticoids and glucocorticoids. Likewise, BMP-6 treatment enhanced aldosterone production by upregulating the mRNA expression of StAR, P450scc, and CYP11B2 among the steroidogenic enzymes by H295R cells [27,28]. These findings suggested that both orexin and BMP-6 individually stimulate the expression of steroidogenic enzymes and subsequent adrenal hormone secretion. However, these stimulatory effects on adrenocortical steroidogenesis interact with each other and their effects are modulated via the crosstalk of intracellular signaling.

Regarding the signaling mechanisms, it was revealed that the effects of orexins on adrenocortical steroidogenesis were regulated by OX1R-induced MAPK activation: G_q_- and to a lesser extent G_s_-mediated extracellular receptor kinase 1/2 (ERK1/2) and p38 activation [43]. Moreover, it was reported that the upregulation of 3β-HSD and cortisol by orexin A was blocked by an AKT antagonist [41]. As for BMP signaling, our group has reported that BMP-6 enhances Ang II-induced aldosterone production via the activation of MAPK signaling by interacting with ALK-2 and -3, and ActRII [27,28,29,30]. In the present study, it was found that orexin A upregulated the expression of FSK-induced steroidogenic enzymes and inhibited BMP signaling by influencing I-Smad and ActRII. These results suggest that orexin and BMP actions lead to the maintenance of adrenocortical steroidogenesis by activating intracellular signaling, a part of which is shared by both of them.

Orexins and orexin receptors are expressed in the adrenal cortex in a species-specific pattern [14]. It has been demonstrated that both OX1R and OX2R are expressed in the human adrenal cortex [10,44]. Regarding the localization of orexin receptors in the human adrenal cortex, OX1R is expressed in all three layers (zona glomerulosa, fasciculata, and reticularis) of the adrenal cortex [37,45]. Mazzocchi et al. revealed that OX2R is expressed in the zona fasciculata and reticularis [37], and Randeva et al. showed that OX2R is expressed in the zona glomerulosa and reticularis [46]. As for the physiological roles of the receptors, multiple reports showed that orexin A modulates steroidogenesis via OX1R rather than OX2R in human adrenocortical cells, which was proven by the selective blockade of OX1R [10,41,43]. In the present study, it was elucidated that both OX1R and OX2R were expressed in H295R cells and that BMP-6 treatment inhibited the expression of OX1R. It is thought that the effects of orexin via OX1R are modulated by the local BMP system in adrenocortical cells.

It has been revealed that BMPs, as well as orexin, are among the important accelerators of adrenocortical steroidogenesis and are functionally involved in the control of blood pressure. In our previous studies, it was revealed that BMP-6 enhances Ang II-induced aldosterone production via activation of MAPK signaling in human adrenocortical cells [27,28] and in the rat adrenal gland [47]. It is of note that endogenous BMP-6 [29,47] plays functional roles and that BMP-6 in the adrenal cortex may contribute to the phenomenon of aldosterone breakthrough, in which aldosterone concentration increases above pretreated levels after long-term therapy with an angiotensin-converting enzyme (ACE) inhibitor or an Ang II type 1 receptor blocker (ARB) [30]. Moreover, Farnworth et al. revealed that inhibin A, another member of the TGF-β superfamily, antagonized the inhibitory effects of BMP-6 on CYP17 expression and 17α-hydroxyprogesterone production in mouse adrenocortical cells [48], which indicates that adrenocortical cells have an intrinsic regulatory system of endogenous BMP-6 function. These results indicate that not only orexin but also BMP-6 are functionally involved in the regulation of adrenocortical steroidogenesis and the control of blood pressure. The results of the present study explain the functional interactions of these stimulants of adrenocortical steroidogenesis and may lead to a further understanding of the regulatory mechanism of hypertension.

It has been revealed that orexin also plays physiological roles in the HPA axis. Orexin receptors are expressed at all levels of the HPA axis, including corticotropin-releasing hormone (CRH)-synthesizing neurons in the parvocellular part of the paraventricular nucleus (PVH), corticotrope cells in the pituitary, and also in the adrenal cortex [12]. Central injection of orexins increases the mRNA expression of CRH and arginine vasopressin (ADH) in CRH-synthesizing parvocellular neurons, and the increase in expression is accompanied by increases in circulating levels of ACTH and glucocorticoid [5,49,50]. Given that the central effect of orexins was inhibited by pretreatment with a CRH antagonist [50], it is presumed that orexin activates hypothalamic CRH-producing neurons and the subsequent activation of the HPA axis. In addition, it has been reported that orexin enhances basal but not ACTH-stimulated glucocorticoid secretion and that antagonizing the ACTH receptor blunted the corticosterone response to ACTH but not to orexin [37]. Therefore, it is also possible that orexin has secretagogue effects on adrenal steroids independent of its effects on the HPA axis. Moreover, in our previous study, it was found that BMP-4 suppressed ACTH secretion and that orexin A enhanced pro-opiomelanocortin (POMC) transcription by upregulating CRH receptor signaling and downregulating BMP-Smad signaling [51,52,53]. Taken together, the results indicate that orexin and the BMP system mutually activate adrenocortical steroidogenesis both directly and indirectly by influencing multiple levels in the HPA axis.

Collectively, the results of the present study indicate that orexin stimulates adrenocortical steroidogenesis in FSK-free and FSK-induced conditions and that orexin and BMP-6 modulate steroidogenesis by suppressing each other’s downsignaling in the adrenal cortex. It was shown that orexin and the BMP system control adrenocortical steroidogenesis directly and via the activation of the HPA axis. Further research on the functional interaction between orexins and the endogenous BMP system in the adrenal cortex could expand our understanding of the pathophysiology of the secretory regulation of adrenocortical steroids.

## 4. Materials and Methods

### 4.1. Experimental Reagents

Forskolin (FSK) was purchased from Sigma-Aldrich Co. Ltd. (St. Louis, MO, USA) and recombinant human BMP-6 was purchased from R&D Systems Inc. (Minneapolis, MN, USA). Human orexin A was purchased from Wako Pure Chemical Industries, Ltd. (Osaka, Japan). The H295R human adrenocortical cell line was cultured in DMEM/F12 containing 10% FBS supplemented with insulin-transferrin-selenium (ITS-G; Thermo Fisher Scientific, Waltham, MA, USA) with 5% CO_2_ at 37 °C [27].

### 4.2. Quantitative Real-Time PCR Analysis

H295R cells (3 × 10^5^ cells/mL) were treated with FSK (1 μM), BMP-6 (30 ng/mL), and orexin A (10 to 300 nM) in serum-free DMEM/F12 in 12-well plates for 24 h. Total RNAs were extracted using TRI Reagent^®^ (Cosmo Bio Co., Ltd., Tokyo, Japan) and then RNA concentrations were evaluated using the NanoDrop^TM^ One spectrophotometer (Thermo Fisher Scientific) and the LightCycler^®^ 96 system (Roche Diagnostic Co., Tokyo, Japan). Primer pairs for PCR were determined from different exons to eliminate PCR products derived from chromosomal DNA. Primer pairs for steroidogenic acute regulatory protein (StAR), inhibitor of DNA binding 1 (Id-1), Smad6, Smad7, activin receptor-like kinase (ALK)-2 and -3, activin type-II receptor (ActRII), BMP type-II receptor (BMPRII), and ribosomal protein L19 (RPL19), a housekeeping gene, were prepared as we reported previously [54]. Primer pairs for cytochrome P450 family 11 subfamily B member 2 (CYP11B2), cytochrome P450 family 17 subfamily A member 1 (CYP17), and 3β-hydroxysteroid dehydrogenase type 1 (HSD3B1) were prepared according to other reports [31,33]. Other primer pairs were prepared as follows: OX1R, 214–233 and 352–371 (from GenBank accession #NM_001525); OX2R, 113–132 and 297–316 (NM_001384272); BMP-6, 1760–1779 and 1912–1931 (NM_00718). Reverse transcription was performed using ReverTra Ace^®^ (TOYOBO CO., LTD., Osaka, Japan), and quantitative PCR (qPCR) analysis was then performed using the MyGo Pro qPCR Instrument (IT-IS Life Science Ltd., Dublin, Ireland) after optimizing the annealing conditions. The relative mRNA expression of the target genes was evaluated by using the Δ threshold cycle (Ct) method. The values of ΔCt were calculated by subtracting the Ct values of RPL19 from those of the target genes. Each target mRNA level, normalized to the RPL19 mRNA level, was expressed as 2^−(ΔΔCt)^. The results are shown as ratios of target mRNA to RPL19 mRNA.

### 4.3. Western Immunoblotting Analysis 

H295R cells (3 × 10^5^ cells/mL) were pretreated with orexin A (300 nM) in serum-free DMEM/F12 for 24 h. After stimulation with BMP-6 (30 ng/mL) for 1 h, the cells were solubilized using a sonicator in 100 μL RIPA lysis buffer (Upstate Biotechnology, Lake Placid, NY, USA) containing 1 mM Na_3_VO_4_, 1 mM NaF, 2% SDS, and 4% β-mercaptoethanol. The cell lysates were then subjected to SDS-PAGE/immunoblotting analysis using antibodies against phospho-Smad1/5/9 (pSmad1/5/9) and total-Smad1 (tSmad1; Cell Signaling Technology, Inc., Beverly, MA, USA). The integrated signal density of each protein was analyzed using the C-DiGit^®^ Blot Scanner System (LI-COR Biosciences, Lincoln, NE, USA). To evaluate phospho-Smad1/5/9 levels, the ratios of the signal intensities of phospho-Smad1/5/9/total-Smad1 were calculated.

### 4.4. Statistics

All data were obtained from at least three independent experiments performed in triplicate. All of the results are shown as means ± SEM. Statistical analysis was performed using an unpaired *t*-test. The *p* values < 0.05 were accepted as statistically significant.

## Data Availability

Data is contained within the article.

## Abbreviations

ACTH, adrenocorticotropin; ActRII, activin type-II receptor; ALK, activin receptor-like kinase; Ang II, angiotensin II; BMP, bone morphogenetic protein; BMPRII, BMP type-II receptor; FSK, forskolin; HPA, hypothalamic-pituitary-adrenal; HSD3B1, 3β-hydroxysteroid dehydrogenase type 1; Id-1, inhibitor of DNA binding 1; ORX, orexin A; OX1R, orexin receptor type 1; OX2R, orexin receptor type 2; and StAR, steroidogenic acute regulatory protein.
